# Three-dimensionally printed biphasic calcium phosphate blocks with different pore diameters for regeneration in rabbit calvarial defects

**DOI:** 10.1186/s40824-022-00271-9

**Published:** 2022-06-15

**Authors:** Young-Wook Seo, Jin-Young Park, Da-Na Lee, Xiang Jin, Jae-Kook Cha, Jeong-Won Paik, Seong-Ho Choi

**Affiliations:** Department of Periodontology, Research Institute of Periodontal Regeneration, Yonsei University College of Dentistry, 50 Yonsei-ro, Seodaemun-gu, Seoul, 120-752 South Korea

**Keywords:** Animals, Bone regeneration, Pore diameter, Hydroxyapatite, Beta tricalcium phosphate

## Abstract

**Background:**

Biphasic calcium phosphate (BCP) is the most frequently used synthetic bone substitutes, which comprises a combination of hydroxyapatite (HA) and beta-tricalcium phosphate (b-TCP). Thanks to the recent advances in digital dentistry and three-dimensional (3D) printing technology, synthetic block bone substitutes can be customized to fit individual defect morphologies. The diameter of the pores can influence the rate of bone formation and material resorption. The aim of this study was to compare three-dimensionally printed biphasic calcium phosphate (BCP) block bone substitutes with different pore diameters (0.8-, 1.0-, and 1.2- mm) for use in the regeneration of rabbit calvarial defects.

**Methods:**

Four circular defects were formed on the calvaria of ten rabbits. Each defect was randomly allocated to one of the following study groups: (i) control group, (ii) 0.8-mm group, (iii) 1.0-mm group, and (iv) 1.2-mm group. All specimens were postoperatively harvested at 2 and 8 weeks, and radiographic and histomorphometric analyses were performed on the samples.

**Results:**

Histologically, the BCP blocks remained unresorbed up to 8 weeks, and new bone formation occurred within the porous structures of the blocks. After the short healing period of 2 weeks, histomorphometric analysis indicated that new bone formation was significantly greater in the BCP groups compared with the control (*p* < 0.05). However, there were no significant differences between the groups with different pore diameters (*p* > 0.05). At 8 weeks, only the 1.0-mm group (3.42 ± 0.48 mm^2^, mean ± standard deviation) presented a significantly larger area of new bone compared with the control (2.26 ± 0.59 mm^2^) (*p* < 0.05). Among the BCP groups, the 1.0- and 1.2-mm groups exhibited significantly larger areas of new bone compared with the 0.8-mm group (3.42 ± 0.48 and 3.04 ± 0.66 vs 1.60 ± 0.70 mm^2^, respectively).

**Conclusions:**

Within the limitations of this study, the BCP block bone substitutes can be applied to bone defects for successful bone regeneration. Future studies should investigate more-challenging defect configurations prior to considering clinical applications.

## Background

Alveolar ridge augmentation is frequently performed on deficient alveolar ridges to facilitate dental implant placement [1–3]. Severe resorption of the bony ridge may occur due to aging or periodontal and periapical pathologies. In this situation, a block bone graft is suggested since the block bone provides structural stability to the augmented site. Autogenous block bone has been considered the gold standard due to its combined osteogenic, osteoinductive, and osteoconductive properties. However, there are also disadvantages including additional surgical procedures for block harvesting, the surgery is technically difficult, and donor-site morbidity might occur [4].

Nowadays, computer-aided design and computer aided manufacturing (CAD/CAM) or three-dimensional (3D) printing technology are frequently applied to produce medical devices. In dentistry, these processes are routinely used for the fabrication of dental prosthesis and surgical templates. The benefits of 3D printing such devices include reduction of time and cost of manufacture. Furthermore, the convenience of fabrication can significantly improve clinical efficiency. Recently, the application of 3D printing has expanded to the production of biomaterials. By doing so, block-type bone graft materials can be customized to fit into individual defects, and characteristics of the biomaterial such as the chemical composition and porosity, can be adjusted.

Biphasic calcium phosphate (BCP) is the most frequently used synthetic bone substitutes, which comprises a combination of hydroxyapatite (HA) and beta-tricalcium phosphate (b-TCP) [5]. Calcium phosphate is the natural chemical constituent of the living bone, and so BCP exhibits excellent biocompatibility. The HA component provides the osteoconductive scaffold for cell migration, vascularization, and new bone formation [6–8], while b-TCP is more biodegradable and is the source of calcium and phosphate ions for bone formation [5].

The presence of interconnected pores in the BCP block is vital since they allow cell infiltration and vascularization [9–12]. The diameter of the pores can influence the rate of bone formation and material resorption. Previous studies have found that larger pores result in more-rapid bone formation[13–15], which is due to greater space for initial bone formation and the larger surface area of the material exposed to osteoclast resorption [5]. However, it can be hypothesized that if the pore diameter exceeds a certain threshold, then the osteoconductivity of BCP will be outweighed by the ingrowth of fibrous tissues into the BCP block combined with biomaterial degradation. Numerous studies have attempted to determine the optimal pore diameter for bone formation, but the results have been inconclusive [14–16].

In this study, BCP-block-type substitutes with pore diameters of 0.8, 1.0, and 1.2 mm were fabricated using 3D printing. Our hypothesis was that synthetic BCP block bone substitute with the largest 1.2-mm pores will facilitate the greatest amount of bone formation when placed in a bone defect. The aim of this study was to compare BCP blocks with different pore diameters for the regeneration of rabbit calvarial defects.

## Methods

### Materials

**Fig. 1:**
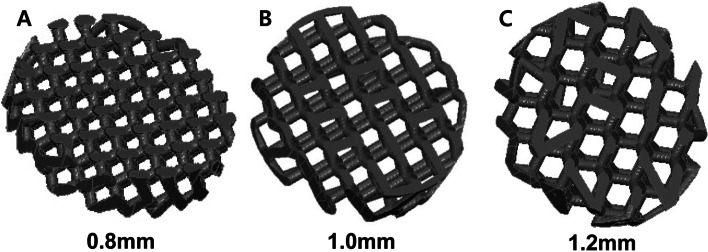
Structural design of the 3D- printed biphasic calcium phosphate (BCP) block bone substitutes. **A** BCP block with 0.8-mm pore diameter; **B** BCP block with 1.0-mm pore diameter; **C** BCP block with 1.2-mm pore diameter

**Fig. 2:**
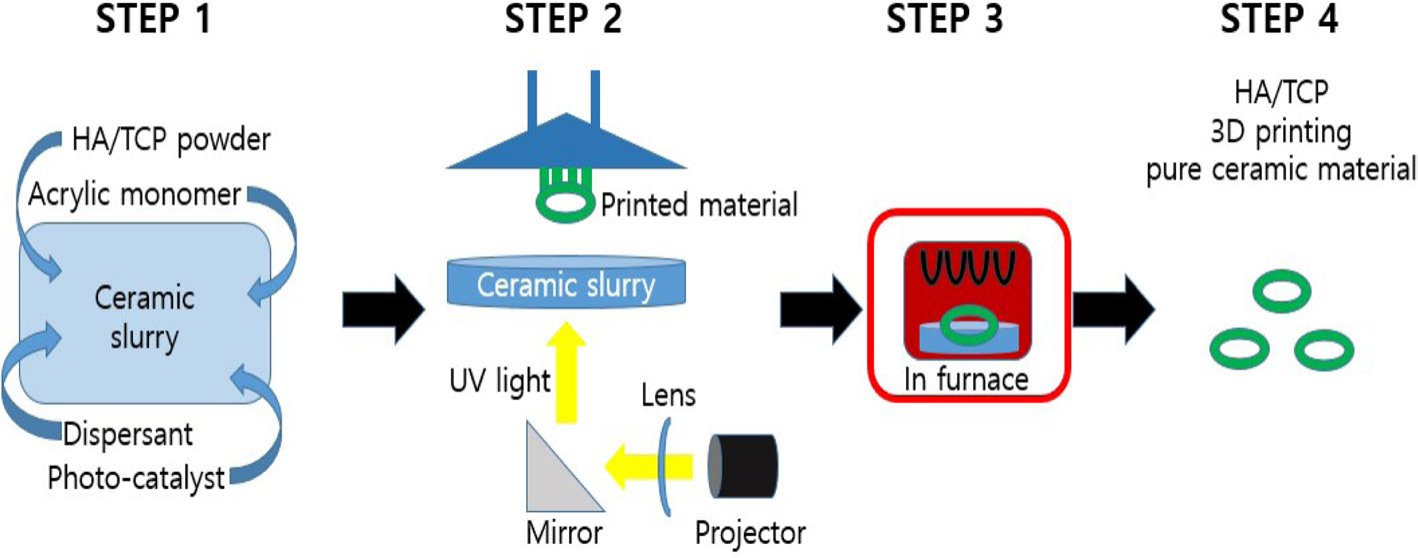
BCP block fabrication process by Digital Light Processing (DLP). **A** Photoreactive ceramic-resin composite composed of hydroxyapatite (HA)/tricalcium phosphate (TCP), acrylic monomers, a dispersant, and a photo-catalyst were mixed in the ceramic slurry. **B** Ultraviolet light was projected to polymerize the slurry and to form the specimen, which was attached to a build plate that slowly moved upward during printing. **C** After the printing was completed, the polymer was completely removed using a heat-treatment step, where the scaffolds were sintered at 1250 °C for 10 h in an electrically heated chamber furnace in ambient air. **D** The polymer residue was removed during sintering by pyrolysis, and the pure BCP block was obtained

**Fig. 3:**
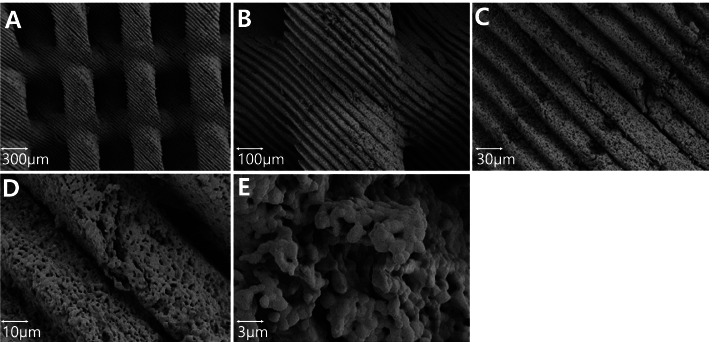
Microstructure surface images of 3D-printed BCP block using scanning electron microscopy (SEM, 3.0 kV) **A** 30x; **B** 100x; **C** 300x; **D** 1,000x; **E** 3,000x. All images showed pure BCP blocks without a residual polymer

This study used 3D printed BCP blocks with various pore diameters. The BCP consisted of HA and b-TCP at a 60:40 ratio, and had pore diameters of 0.8, 1.0, and 1.2 mm. The 3D-printed block bone substitutes were prepared using a digital light processing 3D printer (Cubicon Lux, Cubicon®, Sungnam, Korea) which has a resolution of 20–100 µm and can print layers that are 20–100 µm thick. This 3D printer guarantees an accuracy of 20 µm in the z- axis and 65 µm in the xy- axis. Before production of the specimen, a preliminary sample of 5 × 5x5 cube was printed, sintered and inspected. The horizontal plane of the print was checked at inspection process. All specimens were designed by using computer software program (Materialise 3-Matic, Proto3000®, Ontario, Canada) and manufactured by stacking the BCP layer with the same diamond crystal lattice sphere structure with a thickness of 20 to 100 µm (Fig. 1). The manufacturing process was as follows (Fig. 2): The bone substitute designs (diameter of 8 mm × depth of 2 mm) were first converted into a stereolithography file that was used by the 3D printer to form the bone substitutes in a layer-by-layer manner. Ceramic slurries containing HA/b-TCP powder, acrylic monomer, dispersant, and photocatalyst were prepared. The UV light emitted from the projector of the printer was then reflected by the mirror through the lens on to the ceramic slurry while it was being printed by the build plate. Thereafter, the residual monomers were completely removed using in-furnace heat treatment at 1250 °C for 10 h (Carbolite, Ubstadt-Weiher, Germany) (Fig. 3).

### Animals

Ten New Zealand White rabbits (12 weeks old and weighing 2.8–3.2 kg) were used in the experiments. Animals were housed in separate cages under standard laboratory conditions and diet. The animals underwent an acclimatization period of 1 week prior to the experiments. The housing protocol was based on the guidelines of the Association for Assessment and Accreditation of Laboratory Animal Care International. All of the procedures for animal selection, care, and preparation for anesthesia and surgical procedures followed the protocol approved by the Institutional Animal Care and Use Committee of Yonsei Medical Center, Seoul, Korea.

### Study design

**Fig. 4:**
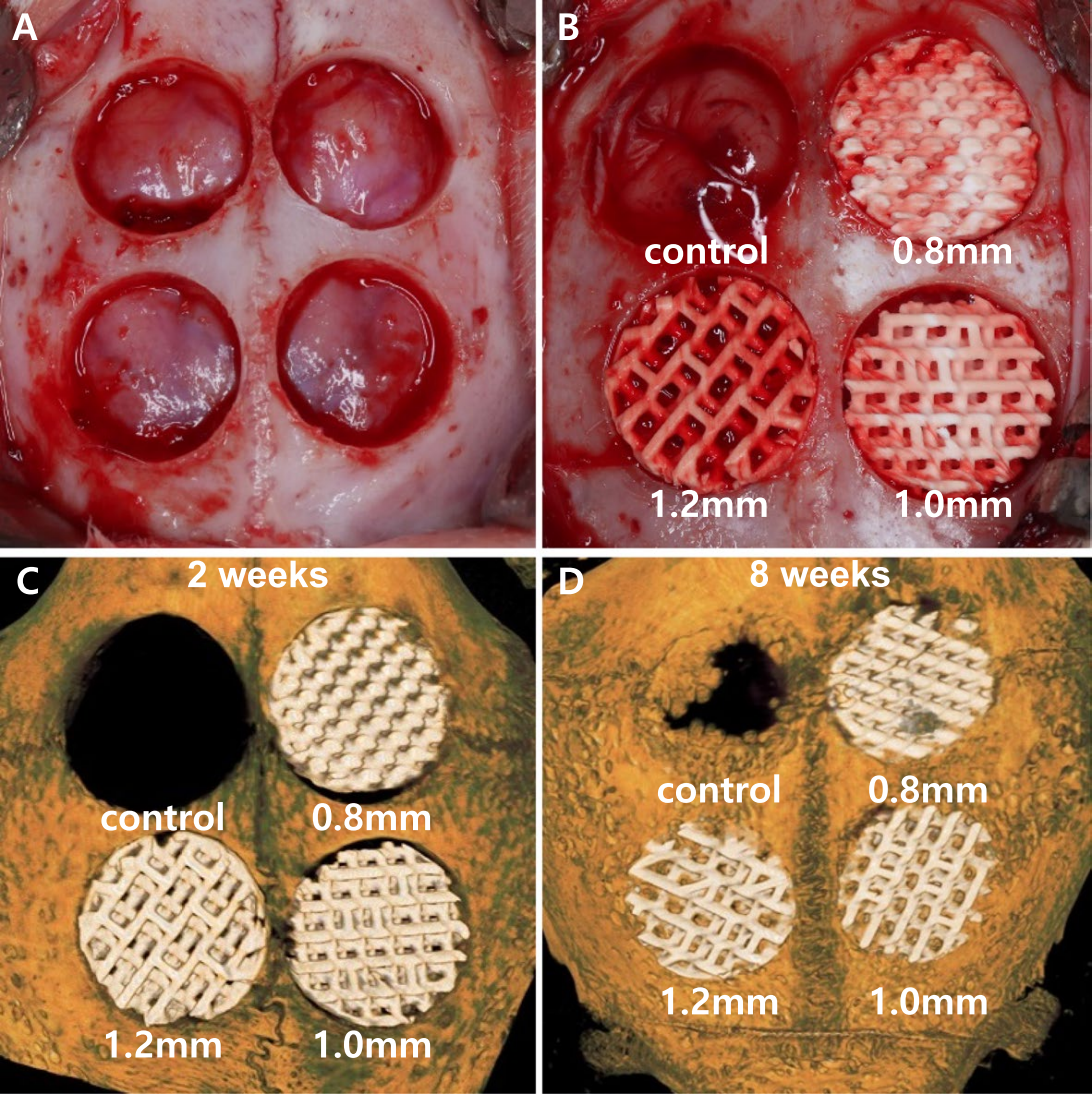
Study design and reconstructed images at 2- and 8- weeks. **A** 4 circular defects were prepared using a trephine bur 8 mm in diameter and 2 mm depth. **B** Each defect was randomly allocated to one of four study groups. Clockwise from upper left: control group, empty; 0.8-mm group, BCP block with 0.8-mm pore diameter; 1.0-mm group, BCP block with 1.0-mm pore diameter; 1.2-mm group, BCP block with 1.2-mm pore diameter. **C** Specimen at 2-weeks (Clockwise from upper left; control group, empty; 0.8-mm group; 1.0-mm group; 1.2-mm group) and **D** specimen at 8-weeks (clockwise from upper left; control group, empty; 0.8-mm group; 1.0-mm group; 1.2-mm group)

Four circular bone defects with diameters of 8 mm were formed on the calvaria of the 10 rabbits. Each defect was randomly allocated to one of the following experimental groups (Fig. 4 A&B):

- (i) control group, empty;
- (i) 0.8-mm group, BCP block with 0.8-mm pore diameter;
- (ii) 1.0-mm group, BCP block with 1.0-mm pore diameter;
- (iv) 1.2-mm group, BCP block with 1.2-mm pore diameter;

All subjects in this study were euthanized at 2 and 8 weeks after the bone graft procedure, and microcomputed tomography (micro-CT) analysis, histomorphometric analysis, and histological observations were performed on the experimental specimens.

### Sample size determination

The sample sized was calculated based on a previous study [17] using a dedicated program (G*Power 3.1.9.4, Germany). For each group, it was estimated that five rabbits would be required under the significance level of 5% and the power of 80%.

### Surgical procedure

A surgical procedure was performed based on those in previously reported study [17]. General anesthesia was performed using isoflurane (2.0–2.5%) inhalation and alfaxan (5 mg/kg) and medetomidine (0.25 mg/kg) intravenous injections. Orotracheal intubation was performed using 6-mm tubes without ballooning to secure the airway. The surgical site was disinfected using povidone-iodine, and local anesthesia was performed using 2% lidocaine with a 1:80,000 epinephrine injection. After making an incision along the cranium midline, a full-thickness flap was elevated and the calvarium was exposed. Four circular defects with a diameter of 8 mm and a depth of 2 mm were created using a trephine bur without damaging the underlying dura mater and cerebral tissue under copious saline irrigation. The defects on the calvarium were randomly assigned to one of the four experimental groups. Each allocated defect was filled according to the study design. After material placement, the flaps were carefully closed and sutured using an absorbable 4–0 sutures (Monosyn, Braun, Terrassa, Spain). General antibiotic therapy using enrofloxacin (10 mg/day) was administered for 5 days after the operation.

### Analysis

#### Clinical observations

Any possible inflammatory signs and unexpected complications of the surgical site were observed each day until the euthanizing at 2 and 8 weeks after surgery. No signs of infection, swelling, inflammation, or wound dehiscence were observed, and no rabbit in this study was lost during the study period.

#### Micro-CT analysis

The calvarial defect specimens (2- and 8-week groups included 10 specimens each) were fixed with 10% formalin for 7 days and then scanned using micro-CT (SkyScan 1173, Bruker CT, Kontich, Belgium) at a pixel size of 13.93 μm (achieved using 130 kV and 60 μA). Scanned data sets were processed in the Digital Imaging and Communications in Medicine format, and reconstructed using 3D reconstruction software (Nrecon reconstruction program version 1.7.0.4, Bruker CT, Kontich, Belgium).

The region of interests (ROIs) for volume measurement in the CT analysis were defined as follows:

- Superior border: the mucoperiosteal layer covering the defect.
- Lateral border: the margins of the original defect.
- Inferior border: the dura meter.

Radiopaque areas were distinguished from the total augmented area using 8-bit threshold grayscale values at a pixel size of 13.93 μm. Grayscale values ranging from 50 to 255 were considered to indicate all mineralized tissue, with those from 50 to 90 considered to indicate newly mineralized tissue in the defects. Values higher than 90 and lower than 50 were considered to indicate BCP material and fibrovascular connective tissue, respectively. Within the ROIs, the following volumes were measured using the software.

- Total augmented volume (TAV; mm^3)^: total volume including fibrovascular connective tissue, newly formed bone, and grafted material volume within the ROIs.
- New bone volume (NBV; mm^3^): sum of newly formed bone volumes in the defect.
- Residual material volume (RMV; mm^3^): residual grafted material volume in the defect.

The proportion of newly regenerated bone was calculated using the following formula.

#### Histomorphometric analysis

After euthanizing the rabbits of each experimental group, tissue fixation was performed in a formalin solution at 4 °C for 1 week. Micro-CT was performed before cutting each calvarial specimen. We prepared 20 tissue specimen slides by cutting the calvarial defect longitudinally. Hematoxylin–eosin was applied to un-decalcified and resin-embedded bone sections to distinguish the mineralized bone matrix from osteoid.

Histological slides were initially scanned using a digital slide scanner (Pannoramic 250 FLASH III, 3DHISTECH, Budapest, Hungary). After microscopic observations of the entire slides including various tissues, the slide images were digitally captured. A slide image analysis program (CaseViewer 2.1, 3DHISTECH) was used for histomorphometric analysis, and the data measured on scanned images were summarized in Excel.

The margins of the ROIs were defined by the defect cut made by the trephine bur. The superior and inferior borders of the ROI were defined by the periosteum and dura mater, respectively. Within the ROI, the following parameters were measured using the software:

- Total augmented area (TAA; mm^2^): total area including fibrovascular connective tissue, newly formed bone, and grafted material volume in the ROI.
- New bone area (NBA; mm^2^): sum of area of newly formed bone volume in the ROI.
- Residual material area (RMA; mm^2^): residual grafted material area in the ROI.

#### Statistical analysis

SPSS software (IBM SPSS Statistics 26, SPSS, Chicago, IL) was used for the statistical analysis. TAV, NBV, and RMV measurements from micro-CT using grayscale and TAA, NBA, and RMA measurements from histomorphometrics and histology were summarized by mean ± standard-deviation values. Kruskal–Wallis and Mann–Whitney U tests were used to analyze the statistic differences among the study groups at each time period (2 and 8 weeks) and between the same groups with different healing periods. Probability values of p < 0.05 were considered statistically significant.

## Results

### Clinical observations

All experimental sites healed uneventfully and were maintained without complications such as infection or wound dehiscence during the study period. At sacrifice, all BCP blocks remained within the grafted site (Fig. 4C and D).

### Micro-CT volumetric analysis

At 2 weeks, NBV was significantly larger in the BCP block 0.8-, 1.0-, and 1.2-mm groups (16.76 ± 3.36, 15.06 ± 2.77, and 16.02 ± 3.61 mm^3^, respectively) than in the control group (6.56 ± 3.53 mm^3^) (*p*  < 0.05) (Table 1), and did not differ significantly between the BCP groups. TAV was also significantly larger in the BCP groups (161.86 ± 8.06, 177.21 ± 26.96, and 177.35 ± 18.40 mm^3^, respectively) than in the control group (*p* < 0.05), also with no significant difference between the BCP groups (*p*  > 0.05). RMV was significantly larger in the 0.8-mm group (67.89 ± 5.75 mm^3^) than in the 1.0-mm (28.24 ± 3.65 mm^3^) and 1.2-mm (31.19 ± 1.24 mm^3^) groups (p < 0.05).

**Table 1:** The results from micro-CT analysis

Healing period	Pore diameter	TAV	NBV	RMV
2 weeks (*n* = 5)	Control (empty)	132.28 [± 12.29]	6.56 [± 3.53]	-
	0.8 mm	161.86 [± 8.06]^(a)^	16.76 [± 3.36]^a^	67.89 [± 5.75]
	1.0 mm	177.21 [± 26.96]^(a)^	15.06 [± 2.77]^a^	28.24 [± 3.65]^(b)^
	1.2 mm	177.35 [± 18.40]^(a)^	16.02 [± 3.61]^a^	31.19 [± 1.24]^(b)^
8 weeks (*n* = 5)	Control (empty)	151.68 [± 16.94]	24.11 [± 1.79]^c^	-
	0.8 mm	189.91 [± 24.60]^(a)^	32.02 [± 3.41]^a,c^	70.53 [± 5.52]
	1.0 mm	170.93 [± 16.61]	35.81 [± 5.73]^a,c^	33.78 [± 2.68]^b,c^
	1.2 mm	190.33 [± 16.60]^(a)^	34.10 [± 5.92]^a,c^	34.69 [± 3.09]^b,c^

*TAV* Total augmented volume, *NBV* New bone volume, *RMV* Residual material volume

^**^Values are presented as mean [± standard deviation] (mm.^3^)

^a^Statistically significant difference compared to the control group.;

^b^Statistically significant difference compared to the 0.8 mm group

^c^Statistically significant difference compared to the corresponding 2-week group at 2 weeks

At 8 weeks, NBV was largest in the 1.0-mm group (35.81 ± 5.73 mm^3^), followed by the 1.2-mm (34.10 ± 5.91 mm^3^), 0.8-mm (32.02 ± 3.41 mm^3^), and control (24.11 ± 1.79 mm^3^) groups. All BCP groups had significantly larger NBVs than the control group, with no significant difference between the BCP groups. There were no significant differences in TAV between the BCP groups, which was larger in the 1.2- and 0.8-mm groups (190.33 ± 16.60 mm^3^ and 189.91 ± 24.60 mm^3^, respectively) than in the control group (151.68 ± 16.94 mm^3^). RMV was significantly larger in the 0.8-mm group (70.53 ± 5.52 mm^3^) than in the 1.0-mm (33.78 ± 2.68 mm^3^) and 1.2-mm (34.69 ± 3.09 mm^3^) groups.

**Fig. 5:**
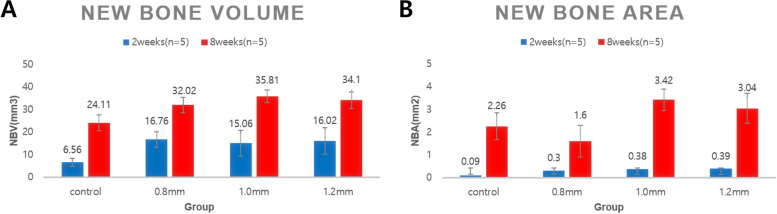
Comparison of (**A**) new bone volume (NBV) by micro-CT analysis and (**B**) new bone area (NBA) by histomorphometric analysis at 2- and 8-weeks. **Values are presented as mean [± standard deviation]

**Fig. 6:**
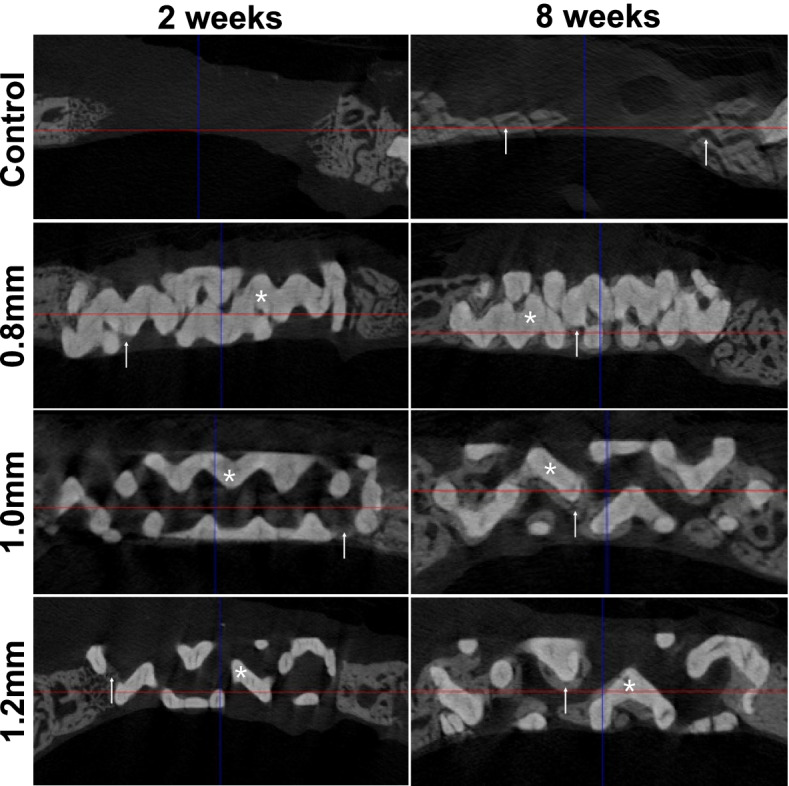
Micro-CT views at 2- and 8- weeks of healing period. White asterisk: biphasic calcium phosphate (BCP) block. White arrow: regenerated new bone

When 2- and 8-week groups were compared, all animals in the 8-week group had significantly larger NBVs than the corresponding 2-week animals (Table 1, Fig. 5A and Fig. 6). TAVs did not differ significantly between the 2- and 8-week groups.

### Histomorphometric analysis

At 2 weeks, NBA was significantly larger in the 0.8-, 1.0-, and 1.2-mm BCP groups (0.30 ± 0.17, 0.38 ± 0.38, and 0.39 ± 0.19 mm^2^, respectively) than in the control group (0.09 ± 0.06 mm^2^) (p < 0.05) (Table 2), and did not differ significant between the BCP groups. TAA was also significantly larger in all BCP groups (15.74 ± 1.95, 15.88 ± 1.00, and 16.83 ± 1.24 mm^2^, respectively) than in the control group (6.13 ± 1.13 mm^2^) (p = 0.008). The 0.8-mm group had the largest RMA (9.61 ± 2.14 mm^2^), but the difference was not statistically significant.

**Table 2:** The results from histomorphometric analysis

Healing period	Pore diameter	TAA	NBA	RMA
2 weeks (*n* = 5)	Control (empty)	6.13 ± 1.13	0.09 ± 0.06	-
	0.8 mm	15.74 ± 1.95^a^	0.30 ± 0.17^a^	9.62 ± 2.13
	1.0 mm	15.88 ± 1.00^a^	0.38 ± 0.38^a^	5.15 ± 1.19
	1.2 mm	16.83 ± 1.24^a^	0.39 ± 0.19^a^	5.51 ± 0.59
8 weeks (*n* = 5)	Control (empty)	5.78 ± 1.10	2.26 ± 0.59^c^	-
	0.8 mm	16.52 ± 0.84^a^	1.60 ± 0.70^c^	11.57 ± 0.81
	1.0 mm	15.85 ± 1.04^a^	3.42 ± 0.48^a,b,c^	5.24 ± 0.14^(b)^
	1.2 mm	15.88 ± 1.29^a^	3.04 ± 0.66^b,c^	4.70 ± 0.59^(b)^

Values are presented as mean [± standard deviation] mm^2^

*TAA* Total augmented area, *NBA* New bone area, *RMA* Residual material area

^a^Significantly greater than the control group

^b^Significantly greater compared to the 0.8 group

^c^Significantly greater compared to the corresponding 2-week group

At 8 weeks, NBA was only significantly larger in the 1.0-mm group (3.42 ± 0.48 mm^2^) than in the control group (2.26 ± 0.59) (p = 0.03). Among the BCP groups, NBA was significantly larger in the 1.0- and 1.2-mm groups than in the 0.8-mm group. TAA was significantly larger in the 0.8-, 1.0-, and 1.2-mm BCP groups (16.52 ± 0.84, 15.85 ± 1.04, and 15.88 ± 1.29 mm^2^, respectively) than in the control group (5.78 ± 1.10 mm^2^) (p < 0.05), with no significant difference between the BCP groups. RMA was significantly larger in the 0.8-mm group (11.57 ± 0.81 mm^2^) than in the 1.0-mm (5.24 ± 0.14, p = 0.016) and 1.2-mm (4.70 ± 0.59, p = 0.008) groups.

**Fig. 7:**
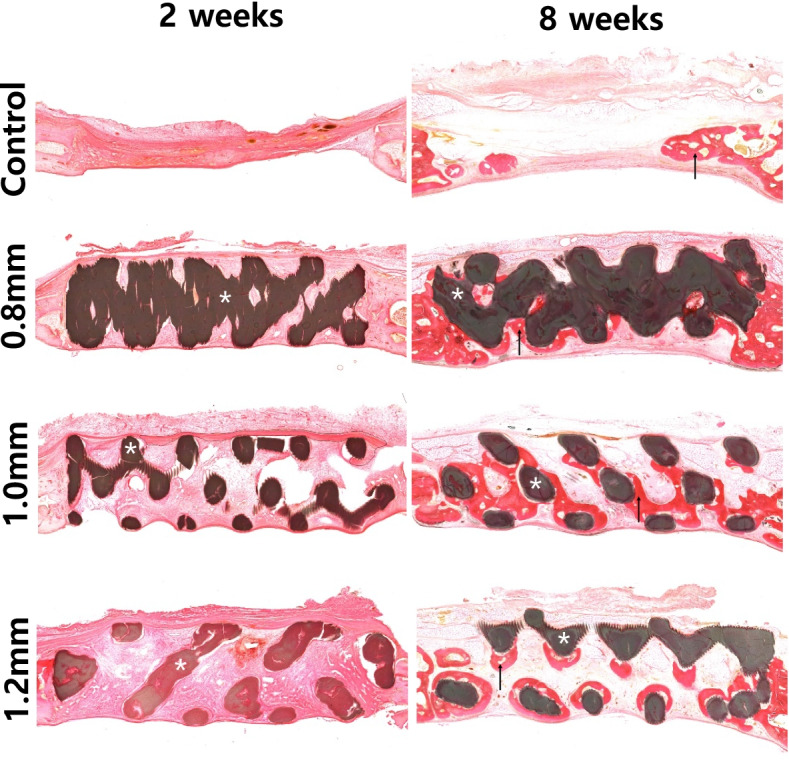
Histomorphometric views at 2- and 8-weeks of healing period. White asterisk: biphasic calcium phosphate (BCP) block, black arrow: regenerated new bone

Comparison of the 2- and 8-week groups revealed that all animals in the 8-week group had significantly larger NBVs than the corresponding 2-week animals in (Table 2, Fig. 5B and Fig. 7). RMA was significantly larger at 8 weeks than at 2 weeks (p < 0.05).

### Histological observations

#### Control group

In the control group, at 2 weeks the defect was partially filled with connective tissue and the center of the defect was sunk down and had a reduced total volume. New bone formation started from the adjacent native bone at the defect periphery (Fig. 7).

At 8 weeks, none of the defects were fully filled with new bone, and some bony islands and bone bridges were observed (Fig. 7).

#### 8-mm group

In the 0.8-mm group, at 2 weeks the volume and shape of the defect were maintained by the BCP block and was completely encapsulated by fibrovascular tissue. The formation of new blood vessels for bone regeneration was observed and the initial bone regeneration started at the calvarial defect periphery (Fig. 7).

At 8 weeks, the BCP blocks remained in place without notable degradation, and new bone formation was observed from the periphery to the center along the surface of the BCP block. However, the new bone had not merged together. New bone formation was also observed in the pore area and mature bone was observed from the periphery of the defect. Overall, remarkable bone regeneration and growth patterns were observed in the BCP block close to the dura meter (Fig. 7).

#### 1.0-mm group

In the 1.0-mm group, at 2 weeks the BCP block maintained the morphology of the formed defect, and an initial healing pattern was observed. Most of the interspaces between the BCP block lattice structure were filled with fibrovascular tissue, but loosely structured tissue was observed in the center of the BCP block. A concave lower boundary due to brain tissue pressure was observed on the dura meter contacting the BCP block (Fig. 7).

At 8 weeks, the 1.0-mm group presented the most new bone formation among the groups using BCP blocks, and bone regeneration occurred on all BCP block surfaces. A ring-shaped bone regeneration pattern was observed surrounding the BCP block surface. The new bone was also fused and matured using a connecting lattice structure pattern in the BCP block (Fig. 7).

#### 1.2-mm group

At 8 weeks, various bone growth types were observed in the 1.2-mm group. Ring-shaped new bone regeneration was observed surrounding the BCP block near to the dura mater (Fig. 7). At the upper part of the BCP block, osteogenesis was observed in a semilunar rather than a ring shape. However, regenerated bone fusion was not observed, indicating an independent bone regeneration pattern including independent island-shape bone formation.

3D-printing technology allows synthetic block bone substitutes to be produced in customized shapes for application in bone augmentation procedures [8, 17–19]. However, the optimal pore diameter within the blocks is yet to be established. The main outcomes of this study were as follows: i) new bone formation was greatest with a pore diameter of 1.0 mm, ii) TAV was maintained in all BCP groups up to 8 weeks, as were the volume of remaining materials, and iii) appositional new bone growth was observed histologically around the lattice structure of the BCP blocks, with almost full defect closure at 8 weeks.

The BCP block bone substitute used in this study had a lattice structure composed of parallel cylindrical rod layers constructed on top of each other in a perpendicular arrangement. Consistent with the results of previous studies, the pore diameter in this study refers to the uniform gap between the rods inside the lattice, and new bone was observed to regenerate in this space [20, 21]. A previous study suggested that the pore diameter of block bone substitutes (HA scaffolds with small [90–120 mm] and large [350 mm] diameters) for enhancing vascularization and new bone formation should be larger than 300 µm [22–24], since pores smaller than 300 µm induced hypoxic conditions that suppressed direct bone regeneration [23, 25, 26]. Consistent with that study, greater bone regeneration was achieved with BCP block substitutes with a larger pore diameter of 500 µm in other studies [14, 15]. In contrast, a previous study using pure b-TCP block substitutes in rabbit calvaria found that bone formation was greater for the smallest pore diameter of 100 µm than for diameters of 250 and 400 µm[16]. However, this result might be explained by the greater biodegradability of b-TCP compared with HA. Considering the lower biodegradability of HA compared with b-TCP, a larger pore diameter with a higher ratio of HA in the mixture of biomaterials is needed [17–19]. The present study used a HA-to-b-TCP at a ratio of 60:40, and the new bone formation was greatest for a pore diameter of 1.0 mm after 8 weeks of healing.

An ideal BCP block would maintain the space of the defect until the defect has been fully regenerated. The remaining material can be biodegraded and be replaced by new bone as long as there is sufficient new bone to facilitate dental implant placements [27, 28]. The optimal degradation rate may vary with the pore diameter, the bone defect configuration, and the individual healing ability of the patient [12, 29]. Nonetheless, the ultimate purpose of designing the best block structure with an adequate pore diameter is to accelerate the bone regeneration process. As indicated by the results of this study, BCP bone substitutes were unresorbed during the 8-week healing period regardless of the pore diameter, as indicated by the maintenance of TAV and the remaining material. Using this biomaterial, it therefore would be reasonable to assume that a larger pore diameter would allow greater bone formation. On the other hand, as the pore diameter increases, the lattice structure density decreases and the compression strength also consequently decreases [30]. The compression strengths, chemical stabilities, and cytotoxicities of BCP blocks with pore diameters of 0.8 to 1.4 mm have been previously reported, which accelerated bone regeneration without unwanted deformation or destruction of the BCP blocks[13]. The BCP block used in the present study promoted bone regeneration without infection or unwanted complications, and exhibited excellent biocompatibility, biodegradability, osteoinductivity, and osteoconductivity. Predictable results can therefore be obtained by applying the customized BCP block using 3D printing to the challenging procedure of reconstructing a wide range of complex bone defects.

The histological analysis of this study revealed that new bone was regenerated along the lattice structure in a BCP block with a pore diameter of 1.0 mm, and osseointegration fused the new bones, indicating accelerated bone regeneration. However, for the 1.2-mm pore diameter, bone regeneration occurred in a ring shape on the lattice structure surrounding the BCP surface, but the connection (fusion) between new bones did not appear and bone regeneration was independent. At the upper part of the BCP block, unlike the lower part adjacent to the dura mater, osteogenesis was observed in a semilunar rather than a ring shape. This might suggest that the dura mater contains greater osteogenic potential compared to the overlying connective tissues. These results differ from those of previous studies, in which larger pore diameters between the lattice structures induced better initial bone regeneration [13], and the critical pore diameter for the BCP block was thought to be between 0.8 and 1.2 mm. In this study, consistent with the findings of the previous studies, when the pore diameter became larger than the critical size, the fibrous tissue penetrated over the defect and bone regeneration was hindered [24, 26]. Further studies are therefore needed to investigate whether the use of a barrier membrane can prevent unwanted fibrotic tissue invasion at the surface of the block and improve the regenerative outcome.

In addition, synthetic BCP blocks are based on HA and beta-TCP, which may enhance new bone regeneration rates in certain physiological conditions. As can be seen from the RMV/RMA results in this study that the volume was maintained during a sufficient healing period and new bone synthesis increased. Predictable results can also be obtained by increasing the suitability and initial stability in the challenging guided bone regeneration (GBR) procedure in the destroyed ridge defect by using the synthetic bone that can be customized using 3D printing. In addition, compared to the particle type, certain block graft types could reduce surgical times, and the risks of postoperative complications including infection, swelling, and morbidity.

In this study, a barrier membrane was not used, so that the effectiveness of the blocks per se could be compared with respect to various pore diameters. Nevertheless, since a barrier membrane might provide cell-occlusion for the blocks with larger pore sizes, the regenerative outcome could be enhanced. Studies using block-type bone graft materials are still insufficient in the literature, and further studies should be performed using different combinations of barrier membranes and block bone grafts.

## Conclusion

In conclusion, within the limitation of this study, BCP block substitutes with different pore diameter promoted faster bone regeneration than that in the natural healing group. In addition, the BCP blocks maintained volume and space for bone regeneration in a sufficient healing period and had greater osteoconductivity and biocompatibility without postoperative complications. Future studies should investigate more-challenging defect configurations prior to considering clinical applications.

## Data Availability

All data generated or analyzed during this study are included in this published article.
